# Nonspecific Gastrointestinal Symptoms as the First Sign of Ganglioneuroblastoma Intermixed—Case Report and Literature Review

**DOI:** 10.3390/jcm12186092

**Published:** 2023-09-21

**Authors:** Łukasz Lipiński, Justyna Lipińska, Maria Kowalczuk, Izabela Kopeć, Magdalena Maria Woźniak, Joanna Mitek-Palusińska, Małgorzata Mitura-Lesiuk

**Affiliations:** 1 Students’ Scientific Society at the Department of Pediatric Radiology, Medical University of Lublin, 20-093 Lublin, Poland; lipinski.lukasz00@gmail.com (Ł.L.); lipinska.justyna98@gmail.com (J.L.); marysia.kowalczuk@gmail.com (M.K.); izabela0kopec@gmail.com (I.K.); 2 Department of Pediatric Radiology, Medical University of Lublin, 20-093 Lublin, Poland; joanna.mp@wp.pl; 3 Department of Pediatric Hematology, Oncology and Transplantation, Medical University of Lublin, 20-093 Lublin, Poland; malgorzata.mitura-lesiuk@umlub.pl

**Keywords:** ganglioneuroblastoma intermixed, high-risk neuroblastoma, abdominal pain, rare tumors

## Abstract

Nonspecific gastrointestinal symptoms remain a problem for pediatricians because, out of a thousand trivial cases, there are rare diseases that require in-depth diagnostics and extensive knowledge to identify them. These complaints may be caused by a neoplastic process. We present the case of a 5-year-old boy whose diagnostic pathway lasted about 3 months. He was admitted to hospital due to severe abdominal pain. Physical examination revealed a bloated, hard, and painful abdomen. In the standing X-ray, the features of intestinal obstruction were visualized. An ultrasound examination showed a possible malignant lesion in the location of the left adrenal gland. After the surgical removal of the pathological mass and histopathological examination, the diagnosis of ganglioneuroblastoma intermixed was made. This tumor, along with neuroblastoma, ganglioneuroma, and ganglioneuroblastoma nodular, belongs to neuroblastic tumors (NTs), which originate from primitive cells of the sympathetic nervous system. NTs are quite rare, but they are still the majority of extracranial solid tumors in children, and their symptoms often appear relatively late when the neoplastic process is already advanced. The purpose of this review is to present current information about ganglioneuroblastoma, with a special emphasis on nonspecific gastrointestinal symptoms as first sign of this tumor and its diagnostics.

## 1. Introduction

Ganglioneuroblastoma intermixed (GNBI) is, in addition to neuroblastoma (NB), ganglioneuroma (GN), and ganglioneuroblastoma nodular (GNBN), one of the four types of neuroblastic tumors (NTs) according to the International Neuroblastoma Pathology Committee (INPC). NTs are the most prevalent extracranial solid tumors in children and originate from primitive cells of the sympathetic nervous system [1]. However, GNBIs generally occur rarely, and more frequently in older children, as opposed to NBs, which are more characteristic of the neonatal and infantile age [2]. GNBs are composed of the intermixing of neuroblastic cells and ganglion cells. They are diagnosed with equal frequency in both sexes, most often in children under 10 years of age [3,4]. This rare neoplasm can manifest with nonspecific gastrointestinal symptoms, which may cause diagnostic challenges among the providers and thus delay the correct diagnosis of the disease.

## 2. The Aim of the Study

We present a clinical case of a child with nonspecific gastrointestinal symptoms as the first sign of GNBI. The aim of this study is to illustrate similar cases, highlight the diagnostic challenges associated with nonspecific gastrointestinal symptoms in children, and present the current state of knowledge about GNBs—its morphology, diagnostic, and treatment options.

## 3. Materials and Methods

The paper was created based on the PubMed and Google Scholar databases. The literature was reviewed using the following key words: ganglioneuroblastoma, high-risk neuroblastoma, abdominal pain, and rare tumors. We also reviewed similar case reports from the last 30 years, where gastrointestinal symptoms were the first sign of GNB in children.

## 4. Case Presentation

A five-year-old male patient presented to the Pediatric Emergency Department (ED) due to severe abdominal pain. Since three months prior to admission to the hospital, the patient had periodically been suffering from constipated stools, which were mostly passed daily, but with increased hardness. Over the past three months, there had been three episodes when the boy did not pass stool for a week. During these times, an increase in paroxysmal abdominal pain was observed. The boy was then treated with trimebutine prescribed by the general practitioner. Three days before his admission to the hospital, the child had passed stool for the last time, and abdominal pain had intensified. In primary care, trimebutine and macrogol therapy were ordered, but due to the lack of improvement, the mother took her child to the ED for the first time. During this visit, the child underwent an examination by a pediatrician and had an enema performed. At that time, the boy’s condition improved, and the child was discharged home. On the next day, the mother and the boy were again admitted to the ED, because the child had developed severe abdominal pain since the morning hours.

**Figure 1:**
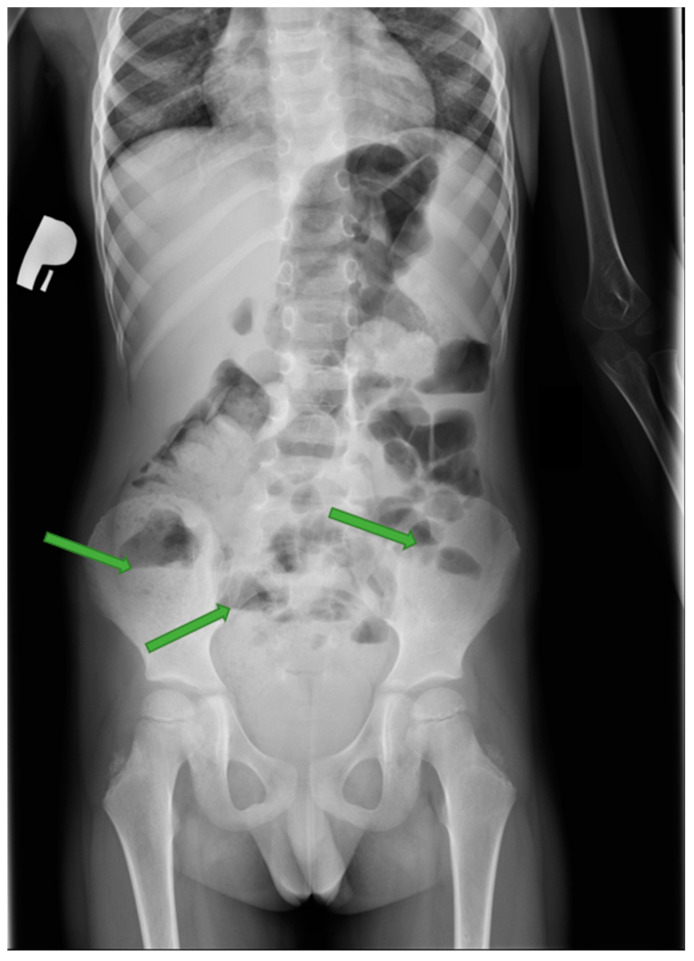
X-ray of the abdomen. Visible multiple levels of fluid in the intestinal loops (green arrows).

**Figure 2:**
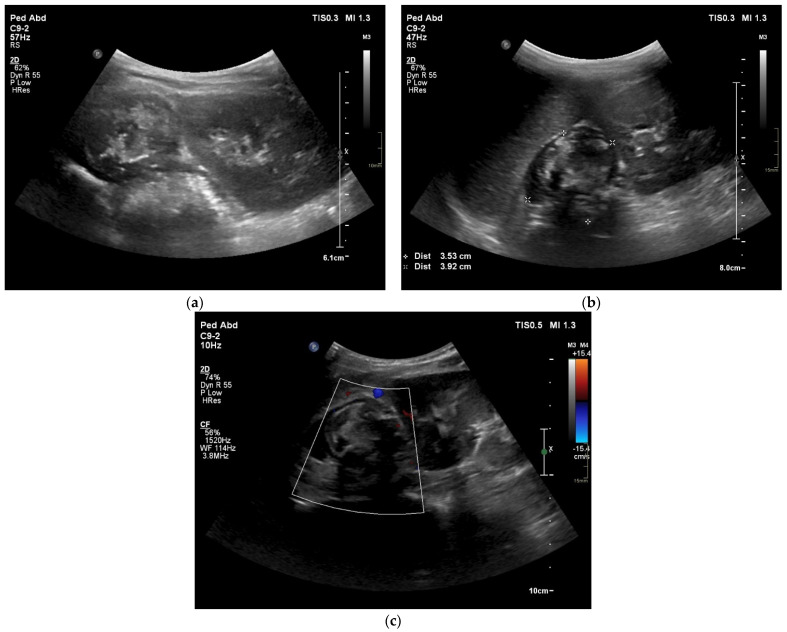
US examination showing (**a**) an echogenically heterogenous area with central hyperechoic reflections above the left kidney, (**b**) modeling of the left upper pole of the left kidney, (**c**) no visible vascular flow signals on Power Doppler.

**Figure 3:**
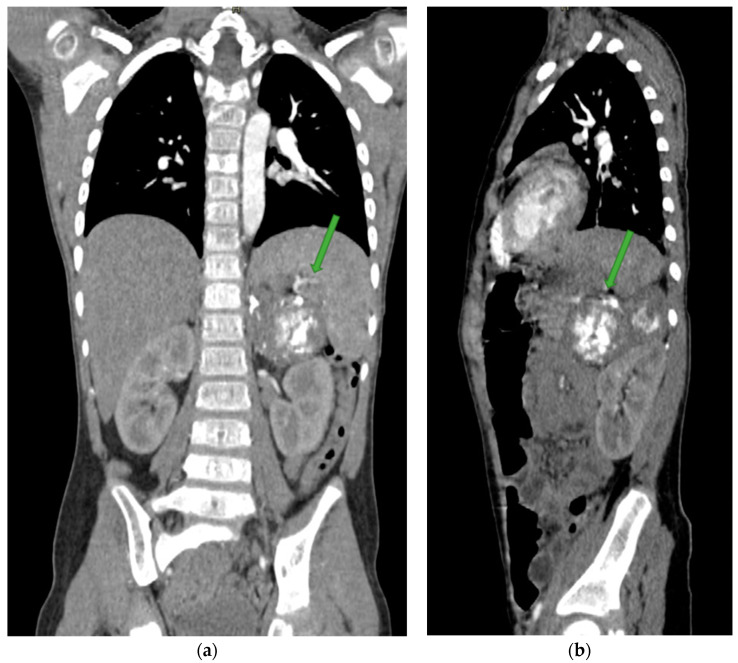
CECT scan of the chest and abdomen showing a visible irregular, heterogeneous lesion within the left hypochondrium (green arrows). (**a**)—frontal section. (**b**)—sagittal section.

**Figure 4:**
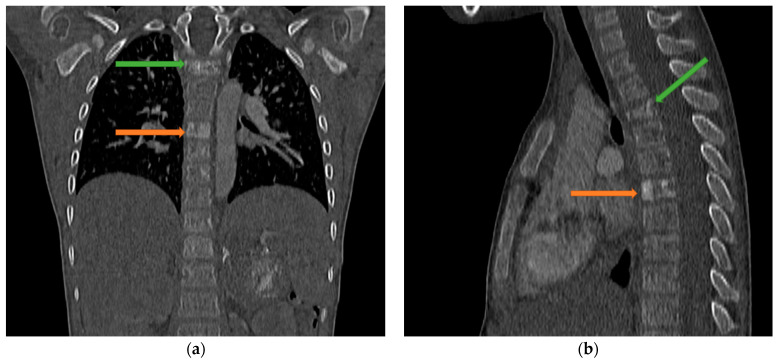
CECT scan of the chest and abdomen showing a sclerotic lesion of T3 (green arrow) on the frontal section (**a**) and a wedge-shaped deformity of T3 on the sagittal section (**b**) and a sclerotic lesion of T6 (orange arrow).

The patient was in average general condition. Physical examination revealed that the abdomen on palpation was bloated, hard, and painful on the entire surface. A standing abdominal X-ray (Figure 1) was performed, which showed features of intestinal obstruction—levels of fluid in the intestinal loops. The child was also examined by a surgeon, who saw no indication for surgical intervention. A follow-up abdominal US (Figure 2a–c) was performed, which detected, in the location of the left adrenal gland, a pathological mass with hyperechoic reflections causing the remodeling of the upper pole of the left kidney. The results of the laboratory tests are shown in Table 1. Due to a suspected malignant lesion, a contrast-enhanced computed tomography (CECT) (Figure 3 and Figure 4) scan of the abdomen and chest was performed the following day. The radiological report described a focal lesion measuring approximately 49 × 63 × 47 mm in the area of the left adrenal gland, with irregular outlines, heterogeneous density, and very numerous calcifications, without obvious features of renal capsule infiltration. In addition, enlarged lymph nodes, some with small calcifications, were visible in the periaortic region. Furthermore, a wedge-shaped deformity of the T3 vertebra, most likely a pathological fracture in the course of the underlying disease, and the T6 vertebra with sclerotic remodeling were visualized.

Based on imaging studies, a neuroblastoma-like lesion was suspected. The child was transferred to the Department of Surgery for the resection of the tumor and then to the Department of Pediatric Hematology and Oncology with the aim of further diagnostics and treatment. 

The additional laboratory tests (Table 2) showed the following abnormalities: elevated serum neuron-specific enolase (NSE), lactate dehydrogenase (LDH), and ferritin, and elevated catecholamines in the 24 h urine collection. A bone marrow aspiration biopsy from two sites and a trepanobiopsy were also performed, which showed bone marrow involvement via the neoplastic process, while molecular testing showed no amplification of the N-MYC oncogene.

During hospitalization, the boy complained of abdominal pain, accompanied by loose stools and pain in the lower limbs, which required opioid analgesics—nalbufine. Due to the boy’s deteriorating condition, pending the result of the histopathological examination, the decision was made to start chemotherapy. The diagnosis of neuroblastoma was suspected because of the location of the tumor (the left adrenal gland); the patient’s age; the presence of metastatic lesions in the bone marrow and in the T3 and T6; and high NSE, ferritin, and OB. Therefore, the patient was classified as being at high risk of neuroblastoma (HR-NBL), and chemotherapy was started according to the COJEC protocol. The tolerance to chemotherapy was generally good. There was a tendency towards thrombocytopenia and anemia, which required a transfusion of 1 unit of RBC concentrate. The following histopathological result was obtained: ganglioneuroblastoma intermixed with a partial tumor differentiation of paraganglioma with bone marrow, bone, and periaortic lymph node involvement. 

131I-Metaiodobenzylguanide (131I-MIBG) scinthygraphy was performed, which showed two distinct foci of accumulation of 131I-MIBG within the T5 and T6 vertebrae. The patient continued chemotherapy and underwent autologous peripheral blood stem cell transplantation (auto-PBSCT). He was also treated with radiotherapy (21 Gy) and immunochemotherapy with 13-cis-retinoic acid and dinutuximab. In a follow-up scintigraphy study, the regression of previously identified lesions with residual tracer accumulation at T6 was visualized. The boy has been facing complications from intensive treatment: leukopenia (grade IV according to WHO), anemia (grade III according to WHO), toxic liver damage (grade II according to WHO), eating disorder, and infection requiring broad-spectrum antibiotic therapy.

## 5. Discussion

Abdominal pain is one of the most frequent complaints reported by children in pediatric healthcare units. While most of the complaints are relatively mild and are associated with functional gastrointestinal disorders, it is very important to always remember about organic causes of abdominal pain that may be life-threatening [5]. The diagnosis and treatment of a child with abdominal pain continue to be frustrating due to a limited history and, often, a lack of alarm symptoms. Often, the pain experienced by a child in the abdominal area is not related to the gastrointestinal system but finds its source in other systems, including the pulmonary, urinary, reproductive, and hormonal. During a child’s examination, special attention should be paid to alarm symptoms that suggest an organic cause of disease. These symptoms include involuntary weight loss; chronic, severe, or nocturnal diarrhea; delayed puberty; genitourinary tract symptoms; persistent right upper or lower quadrant pain; significant vomiting; deceleration of linear growth; gastrointestinal bleeding; pain awakening the child at night; unexplained fever; dysphagia; and odynophagia [6]. In our case, none of these symptoms occurred; therefore, the patient’s diagnostics were expanded only when the child was admitted to the ED with symptoms of acute abdomen pain. The physician’s task and challenge is to determine in which case the cause may be organic [4,7]. 

In addition to the patient’s history and detailed physical examination, imaging studies are extremely valuable tools in the search for organic causes of abdominal pain. These tests can be helpful in finding a pathological mass, and their primary role is to confirm its presence and recognize prominent imaging characteristics that can narrow the range of differential diagnoses. The preferred initial modality to determine the origin of the mass is US [8]. However, it is crucial to keep in mind the indications for any test ordered by physicians, because, in children who do not report alarm symptoms, abnormalities are found on abdominal US with a frequency of less than 1% [9]. In our case, this was the primary test that guided the diagnosis in the search for a neoplastic cause of the symptoms. Given the patient’s age and the location of the suspicious mass, the diagnostic process should have been focused on identifying the tumor and considering surgical treatment options. For this purpose, a CT scan is essential for tumor staging and preparation for surgical management [8]. By using CT in children, emphasis is placed on the ALARA (“As Low As Reasonably Achievable”) principle, which involves limiting time with radiation sources so that patients receive the lowest possible doses [10].

We reviewed the case reports of GNB in children, where the primary complaints were those from the gastrointestinal system (Table 3).

In the nine case reports, the first sign of GNB was chronic watery diarrhea caused by excessive VIP secretion. All of these children were under 2 years of age, and all complaints were resolved after the surgical removal of the tumor. According to Soga et al., pediatric patients with VIP-secreting GNBs have a good survival rate of 90% [25]. Although chronic diarrhea indicates looking for the cause in the abdomen, the tumor causing VIP secretion can also be located in other locations, such as the mediastinum. Recurring constipation was only noted in three cases, one of which was fatal. In the Jamer et al. case report, as in our case, the child was initially treated ineffectively with macrogol. Imaging studies turned out to be crucial, as they revealed the tumor mass in both instances, confirming the organic cause of the gastrointestinal symptoms.

### 5.1. Epidemiology

The majority of extracranial solid tumors in children and adolescents, comprising 7–10% of all pediatric tumors, are NTs [26,27,28]. NTs are responsible for 12% of deaths associated with cancer in pediatric patients under the age of 15. Some tumors involute spontaneously without treatment, while others progress with fatal results despite the introduction of intensive treatment measures [29]. Due to their wide range of differentiation in terms of morphology and their morphological overlap with other mesenchymal tumors, NTs present a substantial diagnostic challenge [30]. GNBs are rare peripheral NTs that constitute around 20% of all NTs. These tumors exhibit notable cell heterogeneity, with ganglionic cells at different levels of maturation and regions containing calcification [31]. According to Whitlock, the intermixed subtype represents only 3.0% of NT cases [26]. NTs, such as GNBI, typically arise sporadically; however, approximately 1% of cases exhibit a positive family history associated with the disease [32]. Alexander et al. report an average age of onset of the disease of 5–7 years, while in Badiu Tisa et al.’s study, 90% of diagnoses are made under the age of 5. There are very few case reports of GNBI in adults [31]. GNBI can occur in various locations, wherever the cells of the sympathetic nervous system are located [4]. The most common sites of origin are the adrenal medulla, extra-adrenal retroperitoneum, and posterior mediastinum [33,34,35]. Rare localizations of GNBI have also been described, i.e., head, neck, pelvis, lungs, thymus, kidney, anterior mediastinum, or cauda [4,31,36]. Meanwhile, metastases are detected in approximately 50–70% of NB patients at diagnosis, generally via the vascular or lymphatic systems. They usually occur in bone marrow (70.5%) and bone (30.9%). Other rarer locations include the lymph nodes, liver, or brain [30,31,34,37]. In GNBs, metastases frequently occur in locations such as bones, bone marrow, liver (Pepper syndrome), and skin (referred to as ‘blueberry muffin’ syndrome) among individuals under the age of 1. Infrequently, cases of lung and brain metastasis have also been reported [31,38,39,40]. The skull was found to be the most common bone metastatic site in both NB and GNB patients, and a proportion of the patients may develop multiple bone metastatic sites. Referring to the study of He et al., the disseminated tumor was more likely to be seen in children older than 18 months. At the time of diagnosis, our patient had a disseminated disease with the involvement of numerous bone sites, bone marrow, and lymph nodes. The reported incidence of disseminated tumor stage at diagnosis in GNBI and GNBN is, respectively, 13% and 25% [37]. 

### 5.2. Histology and Immunochistochemistry

The histological characteristics of the GNBI subtype encompass small regions of neuroblastic cells at various levels of maturation, the presence of neuropil, and Schwannian stroma constituting more than half of the structure. In comparison to the nodular subtype, GNBIs usually lack hemorrhagic and/or necrotic nodules. Tumors in this category are classified into a Favorable Histology (FH) Group according to The International Neuroblastoma Pathology Classification (INPC) [41]. Immunohistochemically, GNBs, similarly to NBs, are positive for neuronal markers such as neurofilament protein (NFP), synaptophysin, chromogranin, CD56, and NSE. Schwann cells found in GNBs are positive for the S100 protein. However, these markers are not specific and cannot be used as the only diagnostic method [13,30]. According to He et al., serum concentrations of ferritin, LDH, and NSE within the normal range are more commonly found in GNBIs than in NBs [37]. The primary differential diagnoses for GNB consist of GN and NB. Molecular testing to identify MYCN amplification, which is characteristic of NB and is related to a poorer prognosis, may be helpful in distinguishing between these disease entities [3,30]. Our patient lacked these molecular changes, confirming the diagnosis of GNB. However, in the studies by Nezami et al. and Okamatsu et al., it was observed that the amplification of the MYCN gene occurred in, respectively, 4% and 1% of the GNBI patients, all of whom had metastatic disease. Therefore, the diagnoses of GNBI and MYCN amplification are not mutually exclusive [2,41]. According to He et al., most GNBI tumors are localized and present no MYCN amplification, which correlates with higher survival rates [37]. The assessment of tumor progression requires the use of imaging modalities and laboratory tests [42]. Since NTs frequently cause an irregularity in the production, secretion, or catabolism of catecholamines, laboratory testing includes a measurement of catecholamines and their metabolites in blood and urine. [43]. In urine, it is recommended to look for metabolites such as VMA, HVA, and dopamine. The latter can also be found in the blood.

### 5.3. Imaging

Multimodality imaging, including metabolic nuclear imaging, is required for diagnosis, staging, response assessment, and follow-up [44]. US is not recommended for the evaluation of tumors, but due to the widespread availability and safety of this imaging modality, it is used most often as the first test for abdominal symptoms in children, even if a malignant cause is suspected. US findings indicative of NB include internal calcifications (30–90%) and encasement of the vessels. If the tumor originates from the adrenal gland, the nearby kidney may be displaced inferiorly. Often, lymphadenopathy is seen [45]. The primary tumor assessment is conducted using CT and/or magnetic resonance imaging (MRI) [42]. On CECT scans, the characteristics of GNBs display diversity, spanning from well-defined, oblong paravertebral masses with homogeneous enhancement to irregular, cystic, hemorrhagic, or locally invasive masses [33]. Calcifications within the tumor can be seen in up to 50% of cases, similar to our patient, and were evident in the CT scan [13]. It is considered that CT is superior to MRI for surgical planning because it shows the extension of the disease and its venous and arterial vasculature better. Despite CT being generally an excellent imaging modality, according to Swift et al., MRI is preferred because of its intrinsic high-contrast and radiation-free images and its capability to provide additional functional information about the tumor. MRI is preferred over CT in assessing bone marrow disease and chest wall invasion. Specifically, diffusion-weighted imaging (DWI) can offer valuable information as it relates to tumor differentiation. For instance, GNBs show higher apparent diffusion coefficient values as compared to poorly differentiated NBs [44,45]. Mueller et al. found that MRI demonstrates higher sensitivity than metaiodobenzylguanide (MIBG) scintigraphy, whereas the latter has higher specificity. Whole-body MR imaging’s specificity, however, is still insufficient since it is challenging to discern between active disease and treatment response [46]. Therefore, scintigraphy is used to assess the response to therapy. For staging and response assessment in pediatric NBs, two imaging modalities—123 I- and 131-MIBG scintigraphy—are used. MIBG is taken up by norepinephrine transporters, which is demonstrated in up to 90% of NBs [47]. In 32% of children with high-risk NBs and GNBs, follow-up 131I-mIBG scintigraphy after treatment could reveal residual disease that was not identified using diagnostic 123I-mIBG scintigraphy [48]. In contrast, 123I-mIBG is used in the evaluation of bone marrow metastases [44].

### 5.4. Clinical Presentation

GNBs are easily misdiagnosed due to nonspecific symptoms in the early stages of the disease [49]. The clinical manifestation of GNB is directly linked with the primary tumor localization and metastases. He et al. reported that the most typical symptoms found in GNB patients include abdominal pain (68%), abdominal swelling (42%), fever (26%), less frequent vomiting, diarrhea, poor feeding, and a palpable abdominal mass. Additionally, patients may have metastasis-related symptoms such as bone pain, limping, or an enlarged cervical tumor [37]. Children under the age of 2 generally present with a large abdominal mass, fever, and weight loss [36]. The tumors located in the mediastinum can cause stridor and breathing difficulties secondary to pressure on the trachea, and the large tumors in the chest may cause mechanical obstruction leading to superior vena cava syndrome [31]. GNB is also known to produce peptides that may cause paraneoplastic syndromes, including cerebellar encephalopathy, opsoclonus myoclonus, and encephalomyelitis/sensory neuronopathy [21]. Opsoclonus myoclonus syndrome is the most commonly encountered paraneoplastic syndrome, occurring in approximately 2–4% of patients [50]. This neurological condition is characterized by rapid and multidirectional eye movements, involuntary muscle jerks, and ataxia. While patients with this syndrome tend to have a favorable prognosis concerning their underlying tumor, most of them will experience lasting neurological impairments [51]. Wildhaber et al. reported a case of severe constipation as a gastrointestinal paraneoplastic syndrome of GNB. Intestinal pseudo-obstruction as a paraneoplastic syndrome is rare and has mainly been reported in patients with small-cell lung cancers, but only in one Wieldhaber et al. study [21]. Our patient also had symptoms and features of intestinal obstruction on the X-ray.

### 5.5. Treatment

GNBI is widely seen as a malignant tumor that, depending on the stage, requires multimodal therapy [35,52]. The final diagnosis is typically made after a diligent morphological examination of the entire resected tumor [2]. However, for stratification and treatment planning, the International Neuroblastoma Risk Group Staging System (INRGSS) is used, which is based on preoperative imaging [3,53]. It was decided to use the term “image-defined risk factors” (IDRFs) to assess surgical risk based on imaging studies. Therefore, CT or MRI is crucial for imaging primary tumors and metastases and can also be used to assess response to treatment [54]. Prognostic factors such as age older than 18 months, histopathology, and MYCN in amplification pediatric patients are stratified into different risk groups, and on these depend the further proceedings. The low-risk group is mainly treated with surgery; the intermediate-risk group requires surgical management with moderate-intensity chemotherapy; and the high-risk group is treated with a variety of methods, including surgery, chemotherapy, radiotherapy, autologous hematopoietic stem cells, and immunotherapy [55]. In our case, based on the suspicion of metastasis on the CT scan and the child’s age of more than 18 months, the patient was classified as HR-NBL. Surgical resection is the mainstay of treatment for GNBI, while chemotherapy is only occasionally used. According to Yang et al., the overall surgical outcomes of GNBI are favorable, with most having successful macroscopic resections and few complications [1]. The main principle of surgery is to remove the tumor completely while protecting important structures and avoiding functional damage [13]. Chemotherapy after surgical resection is the treatment of choice in metastatic disease, and standard regimens have four main components: induction chemotherapy, local control, consolidation, and maintenance therapy [56,57]. The majority of children with HR-NBL do achieve remission after induction chemotherapy, and due to the ability to deliver induction over a shorter timeframe, the rapid COJEC protocol has been incorporated into the standard treatment [57]. Compared with standard regimens, a rapid induction regimen with an increased dose intensity seemed to improve the 5-year (30.2% vs. 18.2%) event-free survival of patients. Nevertheless, this method of treatment is associated not only with more infectious complications and longer hospital stays but also with late complications, including growth failure, renal dysfunctions, hypothyroidism, hearing impairment, orthopedic impairment, and secondary malignancies [55]. Radiotherapy can also be used in the treatment of HR-NB, mainly to consolidate the locoregional control of residual and relapsed tumors or to treat resistant metastatic tumors. According to Wei et al., the children who received radiotherapy after the surgical removal of the tumor had better outcomes than the nonradiotherapy children’s group. Thus, the efficacy and importance of local radiotherapy for primary lesions are obvious [58]. Maintenance therapy is directed at the eradication of residual disease. Anti-GD2 immunotherapy with dinutuximab is the standard of care. According to Yu et al., the addition of anti-GD2 immunotherapy with dinutuximab improved the 2-year event-free survival rate to 66% [59]. Now, the emerging therapy of 131I MIBG imaging followed by autologous stem cell rescue has shown promising response rates [45].

The International Society of Pediatric Oncology (SIOPEN) conducts trials on NB within Europe. In the current HR-NB protocol, cross-sectional imaging is mandatory for the response assessment of the primary tumor at staging, after induction chemotherapy, for preoperative planning and the evaluation of residual primary tumors after surgery and before radiation therapy, before maintenance, and at the end of treatment [44]. The International Neuroblastoma Response Criteria (INRC) include the use of RECIST (Response Evaluation Criteria in Solid Tumors) guidance for measurable soft-tissue disease combined with nuclear medicine imaging [60]. Nuclear medicine studies, most often MIBG, are used to assess the response of bone marrow metastases. Studies performed by SIOPEN use the SIOPEN score created by them, where the body is divided into 12 skeletal segments and each segment is assessed for disease, with a score of 0 indicating no bone marrow involvement and a score of 6 indicating diffuse NB infiltration of the entire segment. The SIOPEN score has prognostic implications, as patients with a score of >3 after induction chemotherapy have very poor outcomes [61].

### 5.6. Prognosis

The recurrence of GNB occurs mostly in the first 2 years after surgery, and it can be solved by subsequent surgery and chemotherapy [43,56]. Okamatsu et al. reported that the estimated 5-year event-free survival and overall survival rates for GNBI without distant metastases patients stand at 94% and 97%, respectively, following complete tumor resection [41]. According to many studies, the younger the child’s diagnosis age is, the better the survival rates are [56,62,63]. Better prognoses also have NBs that have mediastinal localization. Due to the early onset of the symptoms, patients and their parents seek medical help earlier [31]. However, long-term survival rates for children with HR-NBL, like our patient, are currently around 40–50% in large cooperative group studies [57]. Nezami et al. pointed out in their study that the overall survival (OS) rate in patients with GNB and metastatic disease was much higher than that suggested by other studies (85% at a median of 3.5 years) [2]. Patients with a diagnosis of GNB have a good prognosis, according to Alessi et al. These tumors may regress spontaneously, which occurs in 1–2% of cases, or mature into GNs [36]. 

## 6. Conclusions

Clinicians should be aware of classic manifestations of GNB, especially those caused by disseminated tumors, and must always pay particular attention to the possibility of a neoplastic cause of nonspecific gastrointestinal symptoms. In diagnosing functional gastrointestinal disorders, an organic cause should always be excluded. Imaging studies, such as CT, play a key role in the evaluation of children with nonspecific symptoms, enabling the detection of lesions that are rare and life-threatening. As a result, the likelihood of a timely diagnosis and the early implementation of effective therapeutic protocols increases, ultimately leading to improved survival rates and reducing permanent damage. 

## Figures and Tables

**Table 1:** Laboratory tests on the day of admission to the hospital.

Laboratory Indices	Value	Norm	Laboratory Indices	Value	Norm
Leukocytes (×10^9^/L)	7.99	3.4–9.5	Albumins (g/dL)	4.13	3.8–5.4
Erythrocytes (×10^12^/L)	4.39	4.2–5.2	ALT (U/L)	16.00	0–39
Hemoglobin (g/dL)	11.4	12–14	Amylase (serum) (U/L)	19.00	28–100
Hematocrit (%)	32.9	35–42.4	AST (U/L)	48.00	0–52
MCV (fl)	74.9	76.5–90.6	Total protein (g/dL)	6.69	6–8
MCH (pg)	26.0	25–31	C-reactive protein (CRP) (mg/dL)	1.83	0–0.5
MCHC (g/dL)	34.7	31–35	Bilirubin (mg/dL)	0.37	0.2–1.2
Thrombocytes (×10^9^/L)	221	140–420	LDH (U/L)	819	0–331
PDW (fl)	8.80	12–20	Phosphor (mmol/L)	1.43	1.05–1.8
Neutrophiles (×10^9^/L)	5.54	1.5–7	Gamma-glutamyl-transpeptidase (GGTP) (U/L)	10.00	0–26
(%)	69.3	30–50	Glucose (mg/dL)	63	70–99
Eosinophiles (×10^9^/L)	0.02	0–0.6	TSH (uIU/mL)	1.240	
(%)	0.3	0–7	Creatinine (mg/dL)	0.3	0.32–0.59
Basophiles (×10^9^/L)	0.04	0–0.15	Urine acid (mg/dL)	6.3	1.8–5.5
(%)	0.5	0–2	Lipase (U/L)	13	0–37
Lymphocytes (×10^9^/L)	1.66	1.5–7	Urea (mg/dL)	26.0	15–36
(%)	20.8	30–50	Potassium (mmol/L)	4.07	3.5–5.1
Monocytes (×10^9^/L)	0.73	0.2–1.2	Sodium (mmol/L)	135	132–145
(%)	9.1	2–12	Calcium	2.32	2.2–2.7

**Table 2:** Results of additional tests.

Laboratory Indices	Value	Norm
Ferritin (ng/mL)	344.30	4–67
Homovanilate (HVA) (mg/24 h)	31.23	0–6.9
Vanilymandylate (VMA) (mg/24 h)	53.96	0–3.6
Catecholamines in daily urine collection		
Adrenaline (μg/24 h)	1.70	0.2–10
Dopamine (μg/24 h)	526.78	65–400
LDH (U/I)	556	0–331
NSE (ng/mL)	116.50	0–16.3

**Table 3:** Reported cases of ganglioneuroblastoma in children with primary gastrointestinal complaints, 1993 to 2023.

References	Age of Patient	Assigned Sex	Symptoms	Laboratory Tests	Tumor Description	Treatment	Outcome
Xiu et al. 2023 [11]	4 y	F	Abdominal pain, vomiting for 1 day	Elevated NSE	Left retroperitoneal mass, with very thick blood vessel inside tumor, GNBI, no metastasis	Surgical resection	No recurrence after 1-year follow-up
Jain et al. 2021 [12]	4 y 11 months	M	Distension and intermittent right-sided chest discomfort for 1 year	Negative urinary catecholamines	Right retroperitoneal suprarenal mass, GNBN	Chemotherapy according to HR-NBL1 SIOPEN protocol and surgical resection seven months after starting chemotherapy	No recurrence 2.5 years after diagnosis
Lu et al., 2018 [13]	4 y	F	Progressive inspiratory dyspnea and dysphagia for 1 year	Elevated chromogranin A, S-100 protein and NSE	Mass located in the left oropharyngeal and posterior pharyngeal walls, GNB, no subtype data available	Surgical resection	No recurrence after 1-year follow-up
Jamer et al., 2018 [4]	6 y	F	Chronic abdominal pain for 2 years, recurring constipation	No data available	Right-sided paravertebral tumor with the specific features of neuroblastoma GNBN	Surgical resection	No recurrence
Czkwianianc et al., 2018 [14]	17 months	F	Watery, nonbloody diarrhea, weight loss	Elevated NSE, dopamine, VMA, VIP	Right retroperitoneal mass, atypical GNB	Surgical resection and post-operative chemotherapy	No recurrence 2.5 years after surgery
Czkwianianc et al., 2018 [14]	2 y	F	7-month watery diarrhea history	Increased NSE, dopamine, adrenaline, VMA and VIP serum levels	Retroperitoneal space, GNBI with lymph node involvement	Chemotherapy and surgical resection of tumor and pathologic lymph nodes and post-operative chemotherapy	No recurrence after 2-year follow-up
Prader et al., 2015 [15]	16 months	M	Stagnation of weight gain, abdominal pain, chronic diarrhea, hypersalivation and blepharitis since the introduction of solid food—Eosinophilic Esophagitis as Paraneoplastic Syndrome	Elevated HVA, creatinine, VMA	Paravertebral thoracic mass, anatomically close to the esophagus, GNBI	Surgical resection	At 19 months, total restitution without any signs of eosinophilic esophagitis
Kanık et al., 2014 [16]	15 months	F	Bulky watery diarrhea approximately 10–12 times a day for 4 months	Elevated VIP, urinary metanephrine and VMA	Mass in the right surrenal region, GNB no subtype data available	Surgical resection	No recurrence after 1-year follow-up
González Toro et al., 2013 [17]	20 months	No data available	Chronic diarrhea 5 times a day for 8 months, without vomiting and fever, abdominal bloating, weight loss	Elevated VIP, VMA, dopamine, NSE, and LDH	Paravertebral abdominal mass, GNBI	Surgical resection	At 5 years, no recurrence
Husain et al., 2011 [18]	18 months	F	2-month history of watery, nonbloody diarrhea, vomiting, and abdominal distension	Elevated VIP,	Well-defined mass in the right upper lobe with tracheal shift to the left, GNB, no subtype data available	No data available	No data available
Ito et al., 2005 [19]	4 years	M	Gradually developing abdominal pain, diarrhea, and jaundice	Elevated bilirubin levels, AST, ALT, GGTP, amylase	The mass at the head of pancreas obstructing common bile duct, GNB with lymph nodes around the pancreatic head and contralateral side of aorta involvement, no subtype data available	Chemotherapy and surgical resection of the tumor and paraaortic lymph nodes 6 months after the diagnosis	No data available
Reindl et al., 2005 [20]	13 months	M	For many weeks, watery diarrhea, vomiting, and weight loss	Elevated VIP, VMA, HVA, adrenaline, and noradrenaline	Right paravertebral mass, GNB, no subtype data available	Surgical resection	No data available
Reindl et al., 2005 [20]	14 months	M	Watery diarrhea 6–7 times a day for 10 weeks	Elevated VIP, S-100	Paravertebral mass, GNB, no subtype data available	Surgical resection	No data available
Wildhaber et al., 2003 [21]	19 months	F	Watery diarrhea, 8–9 episodes per day for 4 months, abdominal pain, weight loss	Elevated VIP, VMA, HVA, chromogranin B, NSE	Presacral mass, GNB, no subtype data available	Preoperative chemotherapy according to the German NB-97 study protocol and surgical resection 7-weeks after diagnosis	2 years-post surgery, no recurrence
Wildhaber et al., 2003 [21]	14 y	F	Constipation lasting for several weeks	No abnormalities	Right suprarenal mass, GNB	Surgical resection	Died due to tumor progression
Barbato et al., 2002 [22]	2 y	F	Chronic diarrhea, poor growth, and diagnosed celiac disease	Elevated AGA IgA, AGA IgG, hTG, VIP,	Mass in the right adrenal gland, GNBI	Surgical resection	No recurrence after 1-year follow-up
Somuncu et al., 1996 [23]	12 months	M	Chronic constipation, urinary retention	No abnormalities	Lower abdominal mass	Surgical resection, chemotherapy	No recurrence after 1-year follow-up
Mojtahed et al., 1995 [24]	4.5 y	F	Chronic persistent vomiting and abdominal discomfort for 1.5 years	Elevated HVA	Mass proximal to the bifurcation of the aorta	Surgical resection	No recurrence after 18-month follow-up

## Data Availability

The data that support the findings of this study are available on reasonable request from the corresponding author. The data are not publicly available due to privacy reasons.
